# The Stalk Sign in Bladder Cancer: CEUS Versus MRI

**DOI:** 10.3390/diagnostics16172687

**Published:** 2026-08-22

**Authors:** Fabrizio Urraro, Nicoletta Giordano, Vittorio Patanè, Maria Chiara Brunese, Giuseppe Ambrosio, Anna Russo, Roberto Calbi, Antonio Cioffi, Alfonso Reginelli

**Affiliations:** 1 Department of Life Sciences, Health and Health Professions, Link Campus University, 00165 Rome, Italy; f.urraro@unilink.it; 2 Department of Precision Medicine, University of Campania “Luigi Vanvitelli”, Piazza Luigi Miraglia 2, 80138 Naples, Italy; vittorio.patane@unicampania.it (V.P.); mariachiara.brunese@unicampania.it (M.C.B.); giuseppe.ambr97@gmail.com (G.A.); annarusso81@yahoo.com (A.R.); alfonso.reginelli@unicampania.it (A.R.); 3 Department of Radiology, “F. Miulli” General Hospital, Acquaviva delle Fonti, 70021 Bari, Italy; r.calbi@miulli.it; 4 Department of Urology, Ospedale del Mare, 80147 Napoli, Italy

**Keywords:** bladder cancer, contrast-enhanced ultrasound, fibrovascular stalk, inchworm sign, multiparametric MRI, VI-RADS, muscle invasion

## Abstract

**Background**: The fibrovascular stalk is characteristic of papillary bladder tumors and may be visualized on MRI as the inchworm sign. Contrast-enhanced ultrasound (CEUS) may depict the same structure dynamically by demonstrating pedicle perfusion before tumor enhancement. We hypothesized that CEUS would be more sensitive than the MRI inchworm sign for detecting a histologically confirmed fibrovascular stalk and that stalk presence would be associated with non-muscle-invasive bladder cancer without completely excluding detrusor muscle invasion. **Methods**: This retrospective study assessed 80 consecutive patients with one focal bladder lesion; 69 patients with technically adequate imaging and an adequate TURBT reference standard were included in the final paired analysis. Dedicated retrospective rereads of anonymized stored CEUS cine loops and complete mpMRI datasets were independently performed by two experienced readers per modality, who were blinded to the other imaging modality, histopathology, and each other’s assessments. A blinded pathologist assessed fibrovascular stalk presence and detrusor muscle invasion. Paired performance was evaluated using exact McNemar testing. **Results**: Histopathology identified a stalk in 48 lesions. CEUS detected 46 of 48 stalks, and MRI detected 36, corresponding to sensitivities of 95.8% and 75.0%, specificities of 90.5% and 81.0%, and accuracies of 94.2% and 76.8%, respectively. Interobserver agreement was 94.2% for both signs, with almost-perfect agreement for the CEUS vascular stalk sign (κ = 0.86) and the MRI inchworm sign (κ = 0.88). For MIBC detection, overall CEUS impression and VI-RADS 4–5 showed sensitivities of 66.7% and 87.5% (paired *p* = 0.063), specificities of 88.9% and 68.9% (paired *p* = 0.004), and accuracies of 81.2% and 75.4% (paired *p* = 0.424), respectively. **Conclusions**: However, the two signs are not operationally equivalent because the MRI inchworm sign additionally requires preservation of the underlying muscular layer, a criterion that may have contributed to its lower observed sensitivity. The stalk strongly favored non-muscle-invasive disease but did not exclude detrusor invasion. CEUS provides complementary perfusion information, whereas MRI-based VI-RADS remains central to local staging.

## 1. Introduction

The distinction between non-muscle-invasive bladder cancer (NMIBC) and muscle-invasive bladder cancer (MIBC) determines the initial therapeutic pathway [1,2,3,4]. NMIBC is generally managed with transurethral resection followed by risk-adapted intravesical treatment and surveillance, whereas MIBC may require radical cystectomy, systemic therapy, bladder-preserving multimodal treatment, or a combination of these approaches [5,6,7,8,9]. Histopathology remains definitive, and CEUS findings should not independently determine the NMIBC or MIBC treatment pathway. The potential contribution of CEUS is limited to adjunctive preoperative characterization of an ultrasound-visible lesion and its attachment. Such information may support procedural planning and draw attention to the tumor base and the need for adequate detrusor-muscle sampling during TURBT, but this potential management effect was not evaluated in the present study.

Multiparametric MRI (mpMRI) is the most standardized imaging technique for local bladder tumor assessment. The Vesical Imaging Reporting and Data System (VI-RADS) integrates T2-weighted imaging, diffusion-weighted imaging (DWI), apparent diffusion coefficient (ADC) maps, and dynamic contrast-enhanced (DCE) imaging to estimate the likelihood of detrusor muscle invasion on a five-point scale [10,11,12]. Validation studies and meta-analyses have demonstrated good diagnostic performance, particularly when categories 4 and 5 are considered suspicious for MIBC [13,14,15]. The principal strength of mpMRI is its multiplanar assessment of the tumor base, bladder wall layers, and surrounding tissues [16,17,18,19].

Papillary bladder tumors may be supported by a subepithelial fibrovascular stalk. On MRI, a stalk-like structure separating an exophytic tumor from the muscular layer, often associated with preservation of the low-signal-intensity detrusor, has been described as the stalk or inchworm sign [13,18,20,21,22,23,24]. This morphology usually suggests a non-muscle-invasive growth pattern [20,25,26,27,28]. Nevertheless, a pedunculated papillary component and focal invasion at the tumor attachment may coexist; therefore, the sign is probabilistic rather than an absolute marker of NMIBC [29,30,31,32,33,34,35,36].

Contrast-enhanced ultrasound (CEUS) approaches the same anatomical substrate from a different perspective. Second-generation microbubbles remain intravascular and permit continuous real-time observation of microvascular filling [37,38,39,40]. A perfused pedicle may become visible before enhancement spreads into the tumor, even when its morphology is not clearly resolved on MRI [41,42]. CEUS could therefore demonstrate the vascular pathway supporting a papillary lesion, while MRI primarily characterizes wall architecture and invasive depth [43,44,45,46,47,48].

Previous CEUS studies have mainly addressed bladder tumor detection, global enhancement patterns, and discrimination between NMIBC and MIBC [49,50,51,52,53]. More recently, a structured CEUS-based VI-RADS framework included the vascular stalk among features associated with a low probability of muscle invasion [54]. However, the diagnostic meaning of the CEUS vascular stalk has rarely been tested against the stalk itself at histopathology. Using muscle invasion as the only reference may misclassify a correctly recognized stalk as an imaging error when an exophytic tumor also contains focal muscle invasion [22,23,52]. The primary study hypothesis was that, owing to its continuous real-time depiction of pedicle-first enhancement, CEUS would have greater sensitivity than the MRI inchworm sign for detecting a histologically confirmed fibrovascular stalk. A secondary hypothesis was that histological fibrovascular stalk presence would be associated with NMIBC but would not completely exclude detrusor muscle invasion. Accordingly, the primary objective was to compare the CEUS vascular stalk sign and the MRI inchworm sign using histological fibrovascular stalk presence as the reference standard. Secondary objectives were to evaluate the association between histological stalk presence and muscle invasion and to compare the performance of the overall CEUS impression and MRI-based VI-RADS for identifying MIBC.

## 2. Materials and Methods

### 2.1. Study Design and Population

This single-center study was a retrospective analysis of routinely acquired clinical data generated between March 2025 and April 2026. This interval represents the clinical observation period and not a period of prospective research recruitment. CEUS, mpMRI, and TURBT were performed exclusively as part of standard clinical care and were neither assigned nor modified by the research protocol. The retrospective observational analysis received approval from the local ethics committee on 5 January 2026 (Prot. 154/i/2026), before the initiation of any study-specific activity. The approval authorized the scientific use of eligible routine-care data from the entire prespecified observation period, including examinations performed before the approval date and routine-care examinations subsequently acquired through April 2026. Data generated after 5 January 2026 continued to derive exclusively from standard clinical care and did not constitute prospective study procedures or research enrollment. The dedicated anonymized imaging rereads, histopathological review, research-database construction, and statistical analyses were initiated only after completion of the clinical observation period in April 2026. Written informed consent covering the use of anonymized clinical data and images for research and potential publication was available for all included patients before their data were entered into the research database. The study was conducted in accordance with the Declaration of Helsinki and reported according to STARD 2015 [55].

After completion of the clinical observation period, records from March 2025 to April 2026 were retrospectively screened, identifying 80 consecutive patients with one focal bladder lesion detected at ultrasound. All 80 patients were radiologically eligible and completed conventional ultrasound, CEUS, and VI-RADS-compliant mpMRI on the same day before treatment. No CEUS or mpMRI examination was technically non-assessable. All patients underwent transurethral resection of bladder tumor (TURBT) within two weeks of imaging.

**Figure 1:**
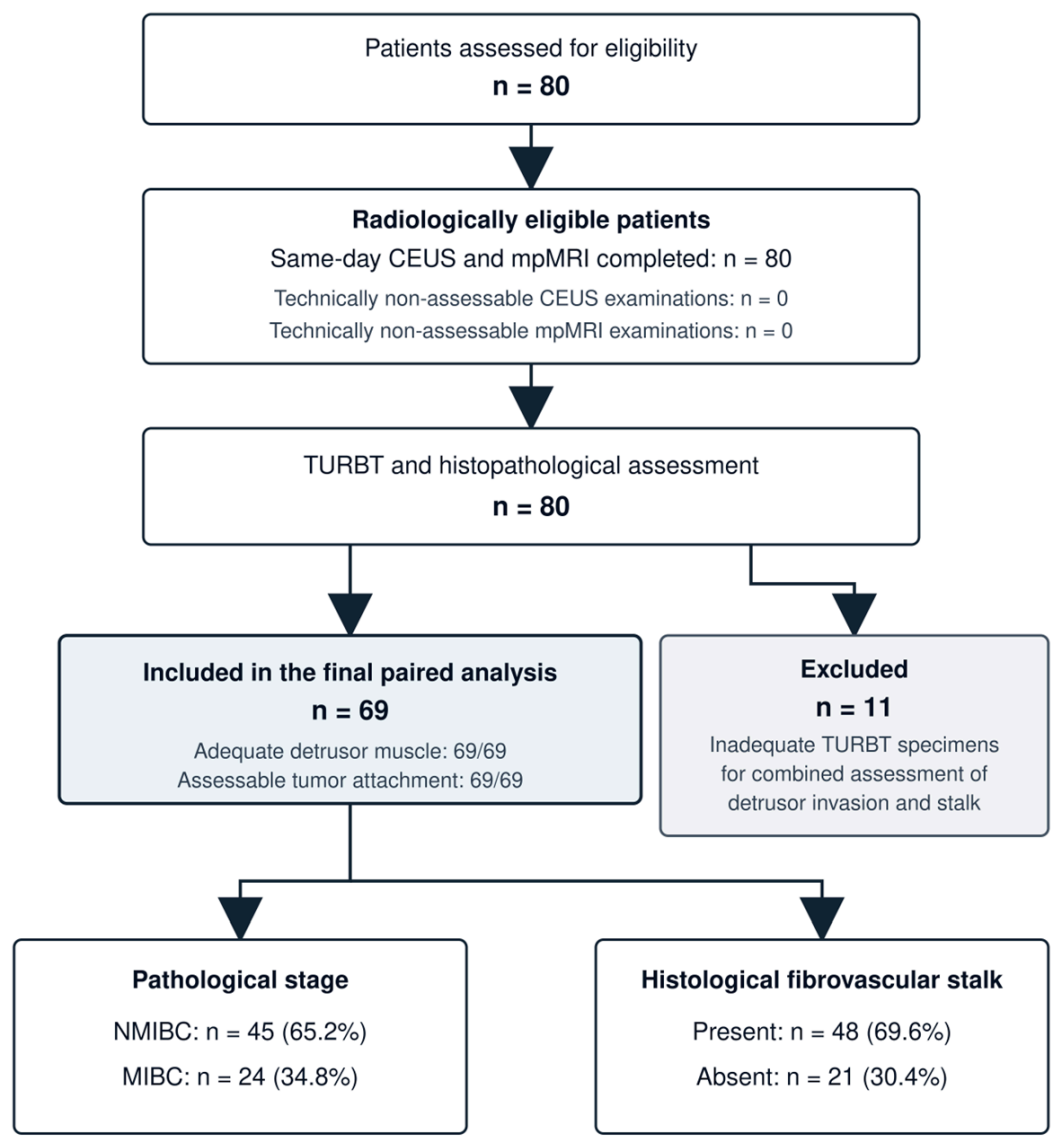
STARD flow diagram of participant selection and inclusion. Eighty consecutive radiologically eligible patients completed same-day CEUS and mpMRI and underwent TURBT. Eleven patients were excluded because the TURBT specimen was inadequate for the combined assessment of detrusor muscle invasion and fibrovascular stalk presence. The final paired analysis included 69 patients with technically assessable CEUS and mpMRI examinations and an adequate pathological reference standard. CEUS, contrast-enhanced ultrasound; mpMRI, multiparametric magnetic resonance imaging; TURBT, transurethral resection of bladder tumor; NMIBC, non-muscle-invasive bladder cancer; MIBC, muscle-invasive bladder cancer.

Inclusion in the final paired analysis required a technically adequate CEUS examination, a complete VI-RADS-compliant mpMRI examination, and a TURBT specimen adequate for the joint assessment of detrusor muscle invasion and fibrovascular stalk presence. A pathological specimen was considered adequate when detrusor muscle was represented sufficiently to assess muscle invasion and the tumor attachment or supporting architecture was represented sufficiently to determine the presence or absence of a fibrovascular stalk. Of the 80 TURBT specimens, 69 (86.3%) fulfilled both adequacy criteria. Eleven specimens (13.8%) were inadequate for the combined pathological assessment and were excluded. No cystectomy specimen was used as the reference standard. Thus, 69 patients with 69 lesions were included in the final paired analysis, with no missing index-test or reference-standard data. One lesion per patient was analyzed to preserve direct lesion-level matching among CEUS, MRI, and histopathology. Participant flow is shown in Figure 1.

### 2.2. US and CEUS Protocol

Ultrasound examinations were performed on a Canon Aplio platform by a radiologist with more than 15 years of experience in genitourinary ultrasound and CEUS. Patients were examined with an adequately distended bladder whenever clinically feasible. Grayscale imaging documented lesion diameter, location, exophytic or sessile morphology, attachment to the bladder wall, and the appearance of the underlying wall. Color Doppler imaging was available as part of the routine examination but was not required for identification of the vascular stalk sign.

CEUS was performed after intravenous administration of 2.5 mL of sulfur hexafluoride microbubbles (SonoVue; Bracco, Milan, Italy), followed by a saline flush. A contrast-specific, low-mechanical-index mode was used. The transducer was positioned to include the lesion, its attachment, and the adjacent bladder wall, and early enhancement was observed continuously. Cine loops were stored for retrospective analysis. The examination focused on the sequence and direction of wash-in rather than on absolute enhancement times, which can vary with cardiac output, injection technique, lesion location, and machine settings.

### 2.3. Definition and Interpretation of the CEUS Vascular Stalk

The CEUS vascular stalk sign was considered present when all of the following qualitative features were observed: a discrete enhancing pedicle extended from the bladder wall into the lesion; enhancement of the pedicle preceded enhancement of the tumor; and contrast propagation could be followed from the pedicle into the tumor during wash-in. Isolated hypervascularity at the lesion base, simultaneous enhancement of the wall and tumor, or a broad area of enhancement without a recognizable pedicle was not considered a positive sign.

At the time of clinical examination, the CEUS radiologist also recorded an overall impression of NMIBC or MIBC. Suspicion of MIBC was based on a broad or irregular tumor base, loss of the normal lesion-wall interface, disruption of wall stratification, enhancement extending into the muscular layer, or extravesical extension. The study classifications were obtained from dedicated retrospective rereads of the stored CEUS cine loops and were not extracted from the original clinical reports. Before review, the cine loops were anonymized and identified using study codes. The first reader was the radiologist who had performed the original CEUS examination, whereas the second reader was a radiologist with more than 10 years of experience in genitourinary imaging who had not participated in the original clinical assessment. Each reader independently recorded the presence or absence of the vascular stalk sign and an overall impression of NMIBC or MIBC. During the study readings, only the CEUS cine loops were available; the readers had no access to MRI, histopathological findings, or each other’s assessments. Their classifications were finalized before linkage with the pathological reference standard.

### 2.4. MRI Acquisition Protocol

MRI was performed on a 1.5 T system (Signa Voyager; GE Healthcare, Milwaukee, WI, USA) using a standardized mpMRI protocol compliant with VI-RADS recommendations. To optimize bladder distension, patients were instructed to drink approximately 500 mL of water before the examination. Antiperistaltic medication was not routinely administered because satisfactory image quality was generally obtained without mandatory antispasmodic medication.

The protocol included high-resolution multiplanar T2-weighted imaging, DWI with ADC maps, and DCE imaging after intravenous gadobutrol at a dose of 0.1 mmol/kg (Bayer SpA, Milan, Italy). T2-weighted imaging comprised axial fat-suppressed PROPELLER (repetition time [TR]/echo time [TE], 9041.0/149.7 ms), sagittal fat-suppressed PROPELLER (8196/140.3 ms), axial non-fat-suppressed PROPELLER (10,509/162.3 ms), and coronal fat-suppressed PROPELLER (9000/164.2 ms), with a slice thickness of 1.5 mm. DWI was acquired using a free-breathing spin-echo echo-planar sequence with spectral fat saturation and b values of 50, 1000, and 1500 s/mm^2^. DCE imaging was performed using a T1-weighted gradient-echo sequence (TR/TE, 5.9/3.1 ms; slice thickness, 1.5 mm) after contrast injection at 1.5–2.0 mL/s. Six acquisitions were obtained at a temporal resolution of 30 s.

### 2.5. MRI Interpretation

The study MRI classifications were obtained from dedicated retrospective rereads of the complete stored mpMRI examinations and were not extracted from the original clinical reports. Before review, all T2-weighted, DWI/ADC, and DCE sequences were anonymized and identified using study codes. The first reader was the radiologist with more than 10 years of genitourinary MRI experience who had originally interpreted the examinations. The second reader was a radiologist with more than 10 years of genitourinary imaging experience who had not participated in the original clinical interpretation. Each reader independently assigned a VI-RADS category from 1 to 5 according to the standard mpMRI framework and separately recorded the MRI inchworm sign as a binary feature. The inchworm-sign classification did not determine the VI-RADS category by itself. The inchworm sign was considered present when an exophytic tumor was elevated from the bladder wall by a visible stalk-like or submucosal component with preservation of the underlying muscular layer, assessed across the complete mpMRI dataset. During the study readings, only the mpMRI examinations were available; the readers had no access to CEUS, histopathological findings, or each other’s classifications. The assessments were performed independently and were finalized before linkage with the pathological reference standard.

### 2.6. Histopathological Reference Standard

The histopathological reference standard consisted exclusively of TURBT specimens; no cystectomy specimen was used. All TURBT procedures were performed using an en bloc resection technique; no conventional piecemeal resection was included. The lesion and its attachment to the bladder wall were submitted as a single specimen for histopathological assessment. All histological slides were reviewed specifically for the study by a pathologist with more than 15 years of experience who was blinded to both imaging assessments. Specimen adequacy was evaluated before classification of the pathological endpoints. A specimen was considered adequate when detrusor muscle was represented sufficiently to assess muscle invasion and the tumor attachment or supporting architecture was represented sufficiently to determine fibrovascular stalk presence. Sixty-nine of the 80 TURBT specimens fulfilled both criteria and were included in the final analysis.

A histological fibrovascular stalk was defined as a discrete, elongated or pedunculated axis of fibroconnective tissue containing identifiable vascular channels, arising from the lamina propria at the tumor attachment and supporting the exophytic papillary tumor. The delicate fibrovascular cores normally present within individual papillary fronds were not sufficient for a positive classification. A broad area of lamina propria, edema, granulation tissue, desmoplastic stroma, or isolated vascular structures at the tumor base without a recognizable supporting pedicle was classified as stalk absent.

The pathologist independently recorded fibrovascular stalk presence and detrusor muscle invasion. The two pathological features were assessed separately because a papillary stalk may coexist with focal invasion at the tumor attachment. Pathological stage was categorized as NMIBC or MIBC according to the presence or absence of detrusor muscle invasion. Tumor grade and histological subtype were not used as reference-standard endpoints, eligibility criteria, or subgroup variables because the study was designed to evaluate the imaging detection of fibrovascular stalk architecture and muscle invasion.

### 2.7. Endpoints and Statistical Analysis

The primary endpoint was the sensitivity of the CEUS vascular stalk sign compared with the MRI inchworm sign for detecting a histologically confirmed fibrovascular stalk. Secondary stalk-related endpoints were specificity, positive predictive value (PPV), negative predictive value (NPV), accuracy, paired concordance, and the association between histological stalk presence and NMIBC. Because this was a retrospective study of a fixed consecutive cohort, no formal a priori sample-size calculation was performed. All eligible patients identified during the predefined study period were considered. Statistical precision was quantified by reporting 95% confidence intervals around the individual diagnostic estimates and the paired performance differences. Unless otherwise specified, the diagnostic-performance estimates were based on the first reader’s dedicated retrospective assessments. The second reader’s classifications were used for the interobserver reproducibility analysis and for the secondary reader-specific performance analysis.

Continuous variables were assessed for distributional normality and are reported as median and interquartile range (IQR) when non-normally distributed. Categorical data were summarized as numbers and percentages. Individual diagnostic proportions were calculated with exact binomial 95% confidence intervals. Differences between paired diagnostic proportions were expressed in percentage points. Their 95% confidence intervals were calculated using the exact melded method of Fay and Lumbard, which provides intervals for paired differences in proportions that are compatible with the central two-sided exact McNemar test. Two-sided central exact McNemar tests compared paired sensitivity among reference-standard-positive lesions, paired specificity among reference-standard-negative lesions, and overall paired accuracy. For stalk detection, CEUS was compared with the MRI inchworm sign. For staging, the overall CEUS impression was compared with VI-RADS 4–5 among the 24 MIBC lesions for sensitivity, among the 45 NMIBC lesions for specificity, and among all 69 lesions for accuracy. Fisher’s exact test evaluated the association between histological stalk presence and NMIBC, and an unadjusted odds ratio with a 95% confidence interval was calculated. Interobserver agreement for binary classifications was quantified using observed agreement and unweighted Cohen’s kappa, calculated as the difference between observed and chance-expected agreement divided by one minus chance-expected agreement. Ordinal agreement for the five-category VI-RADS scale was assessed using linearly weighted Cohen’s kappa, with weights defined as, where. Exact category agreement was calculated as the proportion of cases assigned the same category by both readers, whereas agreement within one category was calculated as the proportion with an absolute interreader category difference of no more than one. For binary ratings, positive agreement was calculated as, and negative agreement as, where and denote concordant positive and negative ratings and and denote the two discordant cells. Bootstrap 95% confidence intervals for the primary kappa estimates were obtained using 10,000 nonparametric patient-level bootstrap replications, resampling the paired reader assessments with replacement; the 2.5th and 97.5th percentiles of the bootstrap distribution were used as confidence limits. Analyses were performed using R version 4.4.2 (R Foundation for Statistical Computing, Vienna, Austria), with the exact 2 × 2 package (version 1.7.0) for exact McNemar tests and compatible paired-difference confidence intervals and the irr package (version 0.84.1) for Cohen’s kappa and linearly weighted kappa. Positive and negative agreement and bootstrap confidence intervals were calculated using custom R functions. All tests were two-sided, and *p* < 0.05 indicated statistical significance.

## 3. Results

### 3.1. Patient and Histopathological Characteristics

Eighty consecutive patients were assessed for eligibility. All 80 were radiologically eligible, completed technically assessable same-day CEUS and mpMRI examinations, and underwent en bloc TURBT; no conventional piecemeal resection was performed. Eleven patients were excluded because the TURBT specimen was inadequate for the combined assessment of detrusor muscle invasion and fibrovascular stalk presence. The final analysis therefore included 69 patients with 69 focal bladder lesions. All 69 included specimens contained adequate detrusor muscle and sufficiently represented the tumor attachment. No cystectomy specimen was used as the pathological reference standard.

Among the 69 included patients, there were 45 men (65.2%) and 24 women (34.8%), and age ranged from 55 to 79 years. Fifty-one patients (73.9%) had a history of smoking, and the mean lesion diameter was 2.1 cm. Among the 69 included patients, there were 45 men (65.2%) and 24 women (34.8%); median age was 67 years (IQR, 66–73 years; range, 55–79 years). Fifty-one patients (73.9%) had a history of smoking, and median lesion diameter was 2.5 cm (IQR, 1.5–4.3 cm). Histopathology classified 45 lesions (65.2%) as NMIBC and 24 (34.8%) as MIBC. The main demographic, clinical, and pathological characteristics of the study population are summarized in Table 1.

A fibrovascular stalk was present at histopathology in 48 lesions (69.6%) and absent in 21 (30.4%). Of the 48 stalk-positive lesions, 43 were NMIBC, and five were MIBC. Of the 21 lesions without a stalk, two were NMIBC, and 19 were MIBC (Table 2).

Thus, 89.6% of stalk-positive lesions were non-muscle-invasive, compared with 9.5% of stalk-negative lesions. Histological stalk presence was strongly associated with NMIBC (odds ratio, 81.7; 95% CI, 14.5–459.2; Fisher exact *p* < 0.001), although the five stalk-positive MIBC lesions demonstrated that the structure did not exclude muscle invasion.

### 3.2. Detection of the Histological Fibrovascular Stalk

**Figure 2:**
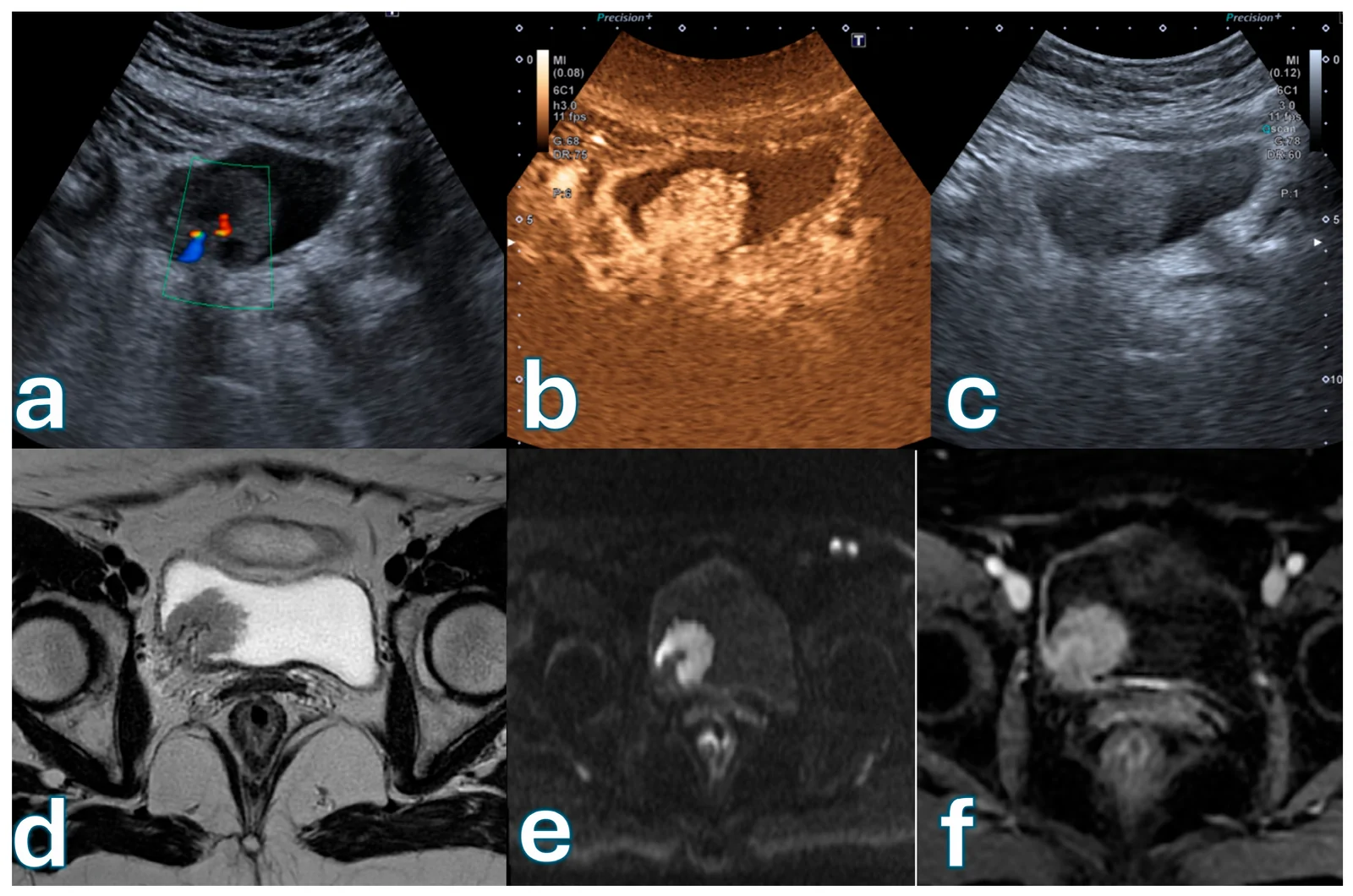
Multimodal imaging of a papillary bladder tumor demonstrating the added value of real-time CEUS for vascular stalk identification. (**a**) Color Doppler ultrasonography shows vascular signals at the tumor base. (**b**) During CEUS, a thin enhancing pedicle is visualized before complete tumor enhancement, with subsequent propagation of contrast from the pedicle into the lesion, fulfilling the definition of the CEUS vascular stalk sign. (**c**) Gray-scale ultrasonography shows the intravesical papillary mass. Corresponding multiparametric MRI images include axial T2-weighted imaging (**d**), diffusion-weighted imaging (**e**), and dynamic contrast-enhanced imaging (**f**). MRI depicts the tumor and its relationship with the bladder wall, whereas the vascular nature of the pedicle is more directly demonstrated by real-time CEUS.

The CEUS vascular stalk sign was positive in 48 lesions. Compared with histopathology, CEUS yielded 46 true-positive, two false-negative, 19 true-negative, and two false-positive results. Sensitivity was 95.8% (95% CI, 85.7–99.5%), specificity 90.5% (69.6–98.8%), PPV 95.8% (85.7–99.5%), NPV 90.5% (69.6–98.8%), and accuracy 94.2% (85.8–98.4%). A representative multimodal imaging case illustrating the vascular stalk sign is shown in Figure 2.

**Figure 3:**
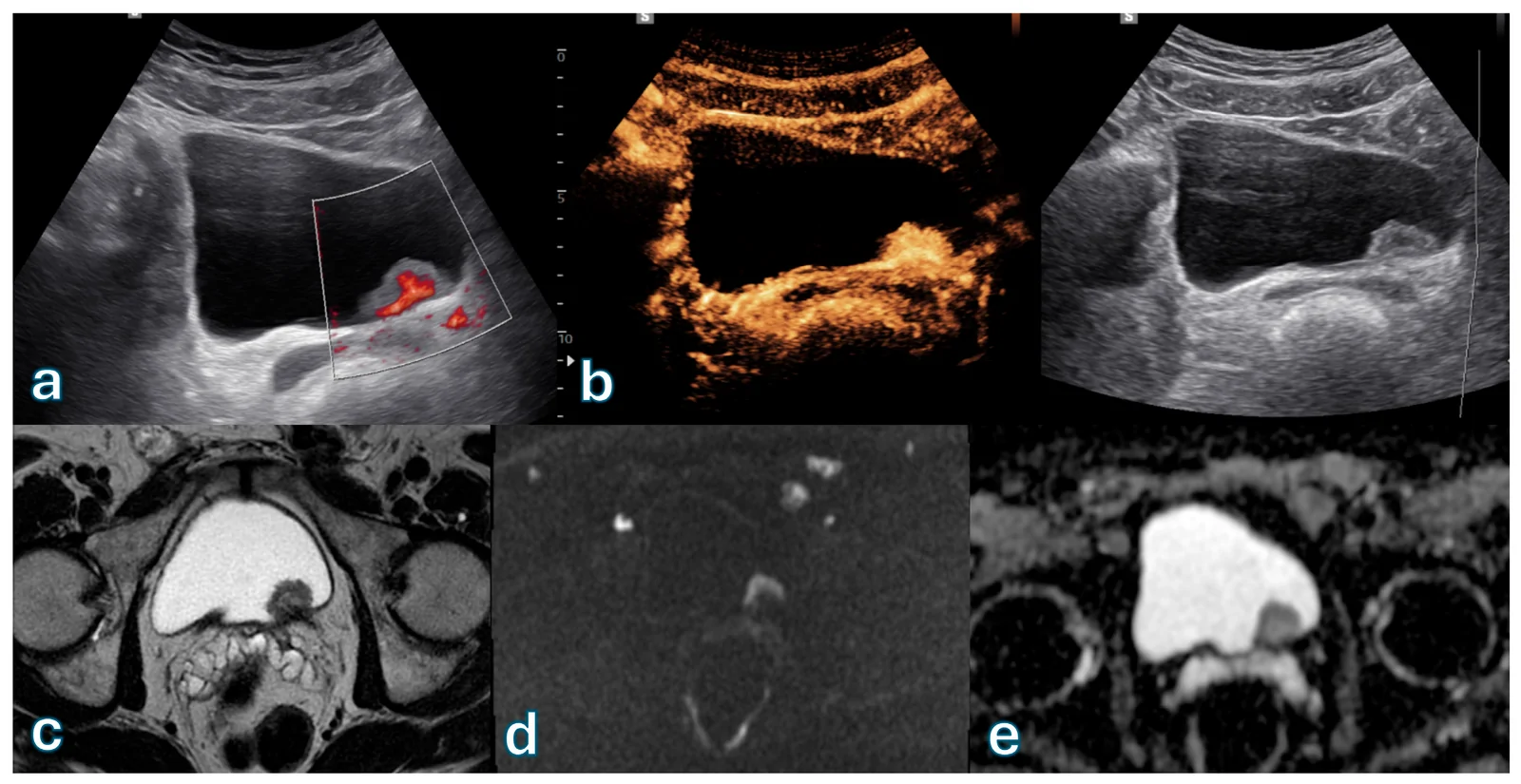
CEUS detection of the vascular stalk in a papillary bladder tumor. (**a**) Color Doppler ultrasonography demonstrates vascular flow at the base of the lesion. (**b**) Split-screen contrast-enhanced and gray-scale ultrasonography shows enhancement of a thin vascular pedicle extending from the bladder wall into the tumor, consistent with a positive CEUS vascular stalk sign. Corresponding multiparametric MRI includes axial T2-weighted imaging (**c**), diffusion-weighted imaging (**d**), and the apparent diffusion coefficient map (**e**). The lesion shows restricted diffusion, with high signal intensity on diffusion-weighted imaging and corresponding low signal intensity on the ADC map. The stalk is more conspicuous on real-time CEUS than on the corresponding MRI images.

The MRI inchworm sign was present in 40 lesions. MRI yielded 36 true-positive, 12 false-negative, 17 true-negative, and four false-positive results. Sensitivity was 75.0% (95% CI, 60.4–86.4%), specificity 81.0% (58.1–94.6%), PPV 90.0% (76.3–97.2%), NPV 58.6% (38.9–76.5%), and accuracy 76.8% (65.1–86.1%). Among the 48 histologically stalk-positive lesions, both imaging signs were positive in 35, CEUS alone was positive in 11, MRI alone was positive in one, and both were negative in one. A representative case in which CEUS demonstrated the vascular stalk despite the absence of a definite MRI inchworm sign is shown in Figure 3.

Compared with MRI, CEUS increased sensitivity for histological stalk detection by 20.8 percentage points (exact compatible 95% CI, +5.0 to +35.9; exact McNemar *p* = 0.006). Among the 21 stalk-negative lesions, both signs were positive in one, CEUS alone was positive in one, MRI alone was positive in three, and both were negative in 16 (Table 3).

**Figure 4:**
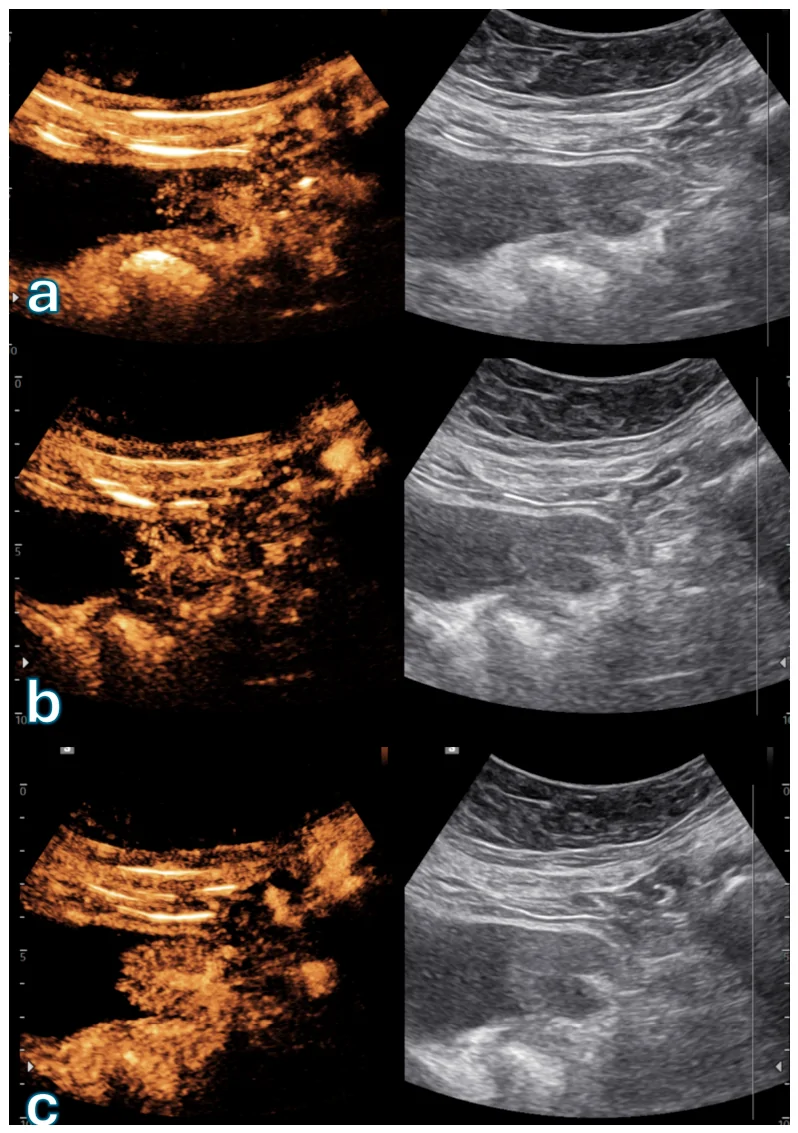
Real-time CEUS demonstration of the vascular stalk sign in a papillary bladder tumor. Split-screen images show contrast-enhanced ultrasonography on the left and the corresponding gray-scale image on the right. Successive frames obtained during contrast wash-in demonstrate initial enhancement of a thin vascular pedicle arising from the bladder wall while the tumor remains largely unenhanced (**a**), followed by progressive propagation of enhancement from the pedicle into the lesion (**b**) and subsequent more diffuse enhancement of the tumor (**c**). This temporal sequence fulfilled the predefined criteria for a positive CEUS vascular stalk sign.

The paired specificity difference was +9.5 percentage points in favor of CEUS (exact compatible 95% CI, −15.1 to +33.6; exact McNemar *p* = 0.625). Across all lesions, CEUS was correct and MRI incorrect in 14 cases, whereas MRI was correct and CEUS incorrect in two. This corresponded to an accuracy difference of +17.4 percentage points in favor of CEUS (exact compatible 95% CI, +4.8 to +29.6; exact McNemar *p* = 0.004). The temporal enhancement pattern used to define the CEUS vascular stalk sign is illustrated in Figure 4.

### 3.3. Assessment of Muscle Invasion

The overall CEUS impression classified 48 lesions as CEUS-NMIBC and 21 as CEUS-MIBC. Histopathology confirmed NMIBC in 40 of the 48 CEUS-NMIBC lesions, while eight were MIBC. Among the 21 CEUS-MIBC lesions, 16 were MIBC, and five were NMIBC. For MIBC detection, CEUS sensitivity was 66.7% (95% CI, 44.7–84.4%), specificity 88.9% (75.9–96.3%), PPV 76.2% (52.8–91.8%), NPV 83.3% (69.8–92.5%), and accuracy 81.2% (69.9–89.6%). The paired performance of the overall CEUS impression and VI-RADS 4–5 for MIBC detection is summarized in Table 4.

VI-RADS scores were two in 24 lesions, three in 10, four in 14, and five in 21; no lesion was assigned category 1. MIBC was found in one of 24 VI-RADS 2 lesions, two of 10 VI-RADS 3 lesions, six of 14 VI-RADS 4 lesions, and 15 of 21 VI-RADS 5 lesions (Table 5).

With VI-RADS 4–5 considered positive, sensitivity for MIBC was 87.5% (95% CI, 67.6–97.3%), specificity 68.9% (53.4–81.8%), PPV 60.0% (42.1–76.1%), NPV 91.2% (76.3–98.1%), and accuracy 75.4% (63.5–84.9%). Among the 24 MIBC lesions, both methods classified 16 as positive, none were positive by CEUS alone, five were positive by VI-RADS alone, and three were negative by both methods. Among the 45 NMIBC lesions, both methods were positive in five, CEUS alone in none, VI-RADS alone in nine, and both were negative in 31. In paired analysis, VI-RADS was numerically more sensitive than CEUS (CEUS−VI-RADS difference, −20.8 percentage points; exact compatible 95% CI, −42.2 to +0.9; exact McNemar *p* = 0.063), whereas CEUS was significantly more specific (+20.0 percentage points; exact compatible 95% CI, +5.1 to +34.6; *p* = 0.004). Overall accuracy did not differ significantly (+5.8 percentage points; exact compatible 95% CI, −6.4 to +18.0; *p* = 0.424). The two approaches therefore showed complementary staging profiles.

### 3.4. Interobserver Reproducibility

All 69 CEUS and MRI examinations were assessable by both readers. For the CEUS vascular stalk sign, the readers agreed in 65 of 69 cases (94.2%). Cohen’s kappa was 0.86 (bootstrap 95% CI, 0.71–0.97), with positive agreement of 95.8% and negative agreement of 90.5%. The second reader obtained 95.8% sensitivity, 90.5% specificity, and 94.2% accuracy for histological stalk detection.

For the MRI inchworm sign, the readers also agreed in 65 of 69 cases (94.2%). Cohen’s kappa was 0.88 (bootstrap 95% CI, 0.76–0.97), with positive agreement of 95.0% and negative agreement of 93.1%. The second reader obtained 75.0% sensitivity, 81.0% specificity, and 76.8% accuracy for histological stalk detection.

Agreement for the overall CEUS classification of NMIBC versus MIBC was almost perfect (κ = 0.85). For the ordinal VI-RADS assessment, exact category agreement was 78.3% (54/69), agreement within one category was 92.8% (64/69), and linearly weighted κ was 0.78. After dichotomization into VI-RADS 1–3 versus 4–5, agreement was also almost perfect (κ = 0.80).

## 4. Discussion

This study demonstrates that real-time CEUS detects the histological fibrovascular stalk in focal bladder tumors more sensitively than the MRI inchworm sign. CEUS identified 46 of 48 histologically confirmed stalks, whereas MRI identified 36. The paired distribution is more informative than the unpaired percentages alone: 11 histological stalks were detected only at CEUS, and only one was detected only at MRI. CEUS also maintained high specificity, with only two false-positive results. The advantage was therefore not explained by indiscriminate labeling of enhancing tissue as a stalk, but by improved recognition of a defined perfusion sequence.

The CEUS vascular stalk sign and the MRI inchworm sign represent the same broad pathological concept but are not equivalent observations. The inchworm sign is primarily morphological. It relies on recognition of a stalk-like or submucosal structure beneath an exophytic tumor and on preservation of the muscular layer across T2-weighted, diffusion-weighted, and contrast-enhanced images. CEUS provides a functional representation: a discrete pedicle enhances first, and microbubble wash-in can then be followed from the pedicle into the tumor. This temporal directionality distinguishes the sign from nonspecific early enhancement, diffuse vascularity, or a broad hyperenhancing tumor base.

Two non-mutually exclusive factors may have contributed to the observed difference in sensitivity. First, CEUS permits continuous real-time observation of microbubble wash-in, allowing direct visualization of the brief interval in which the vascular pedicle enhances before contrast propagates into the tumor. By contrast, DCE-MRI in this study comprised six acquisitions with a temporal resolution of 30 s. Depending on acquisition timing, injection and circulation kinetics, a brief pedicle-to-tumor enhancement sequence may fall between sampled phases, potentially contributing to a false-negative MRI assessment of the stalk. Second, because the MRI inchworm definition also required preservation of the underlying muscular layer, a visible stalk-like component associated with focal muscular disruption could still be classified as an absent inchworm sign. These mechanisms are not mutually exclusive, and the present study cannot quantify their relative contributions. MRI visibility may also be affected by stalk caliber, orientation, motion, and partial-volume averaging. These considerations should therefore be regarded as mechanistic hypotheses rather than proof that either temporal resolution or definitional asymmetry caused the discordant findings. The high PPV of CEUS for a histological stalk is clinically and conceptually important. A positive CEUS sign corresponded to a fibrovascular stalk in 95.8% of lesions. The diagnostic definition probably contributed to this result because positivity required three linked observations: a discrete pedicle, earlier pedicle enhancement, and propagation of enhancement into the tumor. The sign therefore describes a vascular pathway, not merely the presence of blood flow. By comparison, the MRI inchworm sign also had a high PPV of 90.0% but a substantially lower NPV. Absence of the MRI sign did not reliably mean absence of a histological stalk, emphasizing that a negative inchworm sign should not be interpreted as failure of the complete MRI examination or as evidence of invasion by itself. The interobserver analysis supports the reproducibility of both imaging signs among experienced readers. Agreement was almost perfect for the CEUS vascular stalk sign (κ = 0.86) and the MRI inchworm sign (κ = 0.88), and the diagnostic performance obtained by the second reader was consistent with the original analysis. Reproducibility was also strong for CEUS assessment of muscle invasion and VI-RADS classification. Nevertheless, these findings apply to experienced genitourinary readers in a single-center setting. In addition, CEUS agreement was evaluated through retrospective interpretation of stored cine loops and therefore does not establish reproducibility of image acquisition or performance among less-experienced operators.

The pathological findings clarify the relationship between stalk architecture and tumor stage. Histological stalk presence was strongly associated with NMIBC: 43 of 48 stalk-positive lesions were non-muscle-invasive, whereas 19 of 21 stalk-negative lesions were muscle-invasive. This association is consistent with the MRI literature describing stalk and inchworm morphology as a feature of superficial papillary growth. Studies of the inchworm sign have nevertheless reported occasional invasive tumors among sign-positive lesions. Our five stalk-positive MIBC tumors reinforce this limitation. A fibrovascular stalk describes the architecture of the exophytic component, while pathological stage is determined by the deepest invasive focus. A papillary tumor can retain a pedunculated component and simultaneously invade the detrusor at its attachment or elsewhere along the base.

This distinction prevents an important interpretive error. A vascular stalk should increase confidence that a lesion has papillary, perfused architecture, but it should not automatically downgrade a tumor with suspicious disruption of the muscular layer. A CEUS-positive stalk and a VI-RADS 4 or 5 classification are not mutually exclusive. They may describe two coexisting features: an exophytic component supplied by a pedicle and focal invasion at the attachment. The appropriate response to this apparent discordance is careful reassessment of the tumor-wall interface, not rejection of either finding.

This radiologic-pathologic separation is the principal added value of the present study compared with earlier work on the inchworm sign. Most MRI studies have evaluated the sign as a predictor of stage, which is clinically useful but does not directly establish whether the imaged structure corresponds to a fibrovascular stalk [5,6]. Here, the primary reference was the histological stalk itself, and muscle invasion was analyzed independently. The design therefore answers a narrower anatomical question with less circularity: whether each modality actually recognizes the supporting pedicle. The finding that CEUS detected 11 additional histological stalks is relevant even though some of those tumors may still require treatment as MIBC. It identifies a specific capability of real-time perfusion imaging rather than simply reproducing the association between papillary morphology and lower stage.

The secondary staging results also argue against presenting CEUS as a direct replacement for mpMRI. VI-RADS 4–5 identified 21 of 24 MIBC lesions, compared with 16 detected by the overall CEUS impression. The paired sensitivity difference favored VI-RADS by 20.8 percentage points, although the exact paired comparison did not reach statistical significance (*p* = 0.063). Conversely, CEUS correctly classified 40 of 45 NMIBC lesions, compared with 31 correctly classified by VI-RADS, resulting in significantly greater specificity for CEUS (paired difference, +20.0 percentage points; *p* = 0.004). Overall accuracy did not differ significantly between the two approaches (*p* = 0.424). This pattern is compatible with their different strengths: CEUS can demonstrate definite interruption or early enhancement of the wall when the invasive component is conspicuous, whereas VI-RADS integrates morphology, diffusion restriction, enhancement, and muscular-layer continuity and is specifically designed to estimate invasive probability. The conclusion is therefore feature-specific rather than modality-wide. CEUS was superior for the primary endpoint of histological stalk detection; it was not superior for the secondary endpoint of MIBC sensitivity. MRI remains the more comprehensive local staging examination because it assesses the complete bladder wall, perivesical tissues, and the pelvis in multiple planes. CEUS is best understood as a focused dynamic examination that can add information about the attachment and perfusion of a lesion already visible at ultrasound. This complementary model is more clinically defensible than a competition in which one modality must replace the other.

The results are also relevant to the emerging concept of CEUS VI-RADS. Previous CEUS investigations have reported useful performance for bladder tumor detection and staging, although protocols and diagnostic criteria have varied [50,52,53,56]. A recent prospective study by Han et al. developed and validated a five-category CEUS-based VI-RADS system in 193 patients [57]. Vascular stalk presence was included among the major CEUS features associated with lower categories, together with preservation of a continuous hypoenhancing muscular layer and an early hyperenhancing inner layer. In the validation cohort, the reported area under the curve was 0.80 for novice readers and 0.88 for expert readers, with good-to-excellent interreader agreement. A subsequent prospective study also supported CEUS as a useful tool for preoperative assessment of muscle invasion and suggested potential value in conjunction with MRI [58].

Our study does not validate that complete CEUS-based scoring system. We did not assign five CEUS categories prospectively or test all proposed wall-layer features, and the present two-reader analysis should not be regarded as a comprehensive multireader validation of the complete CEUS-based scoring system. Instead, the present work provides direct radiologic-pathologic validation of one biologically meaningful component: the vascular stalk. This is an important added value because the reference condition was the fibrovascular stalk itself, not NMIBC. When muscle invasion alone is used as the reference, a correctly detected stalk in an invasive tumor may be counted as a false-positive low-risk sign. By separating stalk presence from invasion, our analysis shows that CEUS can be anatomically correct even when the lesion is ultimately MIBC.

These findings support further development of a structured CEUS reporting framework, but the term CEUS VI-RADS should be used carefully. VI-RADS is an established mpMRI system with defined acquisition, scoring, and interpretation standards. A CEUS-based analogue requires independent multicenter validation, reproducibility testing, and clear rules for integrating stalk morphology with wall-layer enhancement and extravesical extension. The vascular stalk could be one component of such a system, but it should remain separate from the final judgment of invasion. A structured report might record stalk present, absent, or non-assessable; integrity of the muscular layer; tumor-base morphology; and the overall probability of MIBC.

The present study did not evaluate changes in treatment selection or clinical outcomes; therefore, CEUS findings should not be used independently to assign an NMIBC or MIBC treatment pathway. Its most plausible immediate role is adjunctive problem solving in patients with an ultrasound-visible papillary lesion. When MRI depicts an exophytic tumor but the inchworm sign is absent or uncertain, CEUS demonstration of pedicle-first enhancement may provide additional characterization of a vascularized stalk and the tumor attachment. This information could draw attention to the tumor base during TURBT and reinforce the need for adequate detrusor-muscle sampling. When CEUS findings are suspicious for wall invasion, they may support prompt completion of mpMRI-based local staging, but they should not replace mpMRI or histopathology. Conversely, a positive vascular stalk sign should not lead to treatment de-escalation because stalk architecture may coexist with muscle invasion, as observed in five lesions in this cohort. These potential applications concern diagnostic work-up and procedural planning and require prospective decision-impact evaluation. CEUS may also provide supplementary information when MRI is contraindicated, poorly tolerated, delayed, or unavailable, although the present results do not establish it as a stand-alone staging substitute. The clinically relevant contribution demonstrated by the present study is additional preoperative lesion characterization rather than a proven change in treatment. Identification of a vascular stalk does not eliminate the need for TURBT, because endoscopic resection or biopsy remains necessary for definitive histological diagnosis, grading, and staging. Furthermore, because a stalk may coexist with detrusor invasion, a positive CEUS stalk sign should neither be used to defer TURBT nor automatically downgrade an otherwise suspicious lesion. CEUS instead provides dynamic information about papillary tumor architecture by demonstrating enhancement beginning within a discrete vascular pedicle and subsequently propagating into the tumor. This may increase diagnostic confidence when an ultrasound-visible exophytic lesion has an absent or equivocal MRI inchworm sign or when MRI is contraindicated, poorly tolerated, delayed, or unavailable. Nevertheless, the present study did not evaluate whether CEUS altered surgical planning, treatment selection, or patient outcomes, and it does not establish CEUS as a stand-alone staging method. Its actual incremental management value therefore requires prospective evaluation. The qualitative definition used in this study was intentional. Absolute enhancement times are sensitive to hemodynamics, venous access, injection rate, and equipment. Relative order—the pedicle enhancing before the tumor—may be more transferable across routine examinations. Quantitative measures, such as stalk-to-tumor onset delay, wash-in slope, or time-to-peak, could improve objectivity, but they would require standardized acquisitions and should complement rather than replace recognition of the perfusion pathway. Likewise, the few imaging–pathology discrepancies may reflect very small or unfavorably oriented stalks, incomplete preservation or histological sampling of the tumor attachment, or the lack of exact spatial registration between imaging and histological sections.

The reliability of histological stalk identification also depends on the resection technique and preservation of the tumor–wall interface. Conventional piecemeal TURBT may fragment or thermally alter this interface and interrupt the continuity of a fibrovascular pedicle, making stalk absence less certain. En bloc resection is intended to preserve the lesion, its attachment, and the underlying bladder wall as a continuous specimen and may therefore facilitate assessment of stalk architecture. Comparative studies generally support favorable histopathological quality with en bloc resection, although findings regarding detrusor muscle representation are not entirely uniform, and direct comparative evidence specifically addressing fibrovascular-stalk detection is lacking [59,60]. All procedures in the present cohort were performed en bloc, and 69/80 specimens fulfilled the predefined combined adequacy criteria. Therefore, a sensitivity analysis according to resection technique was not possible, and the present findings should be considered applicable to adequately sampled en bloc specimens rather than automatically generalizable to conventional piecemeal TURBT. The 11 inadequate specimens also indicate that en bloc resection does not invariably guarantee adequate representation of both the tumor attachment and detrusor muscle. Future studies should evaluate decision impact in addition to diagnostic accuracy. Specifically, prospective studies should determine whether adding CEUS to the usual diagnostic work-up changes planned assessment or sampling of the tumor base during TURBT, prompts additional staging examinations, reduces uncertainty in equivocal MRI or VI-RADS 3 cases, or modifies multidisciplinary recommendations. Prospective multicenter cohorts should include independent readers with different experience levels, standardized bladder filling and cine-loop acquisition, documentation of lesion location and stalk caliber, and a predefined category for technically non-assessable cases. Such studies could determine whether a CEUS-based system can serve as a reliable alternative when MRI cannot be performed and whether combined CEUS-mpMRI interpretation performs better than either examination alone.

This study has several limitations. It was retrospective, single-center, and included a relatively small cohort of solitary, focal, ultrasound-visible lesions; the results may not apply to carcinoma in situ, diffuse wall thickening, multifocal disease, or lesions with poor acoustic access. The fixed sample size also limited statistical precision, particularly for stalk specificity, which was based on 21 stalk-negative lesions, and for staging sensitivity, which was based on 24 MIBC lesions. The paired staging comparisons should therefore be interpreted in light of the small numbers of discordant observations. Each modality was evaluated by two experienced readers and demonstrated high interobserver agreement; however, the number of readers remained limited, less-experienced readers were not included, and CEUS agreement reflected interpretation of stored cine loops rather than reproducibility of image acquisition. Because the first CEUS and MRI readers had also performed or initially interpreted the corresponding clinical examinations, recall effects cannot be entirely excluded despite anonymization, independent retrospective rereading, and blinding to the other modality and histopathological findings. Finally, the vascular stalk assessment was qualitative and exact spatial registration between imaging and histological sections was unavailable. Pathological stage was analyzed according to the prespecified clinically relevant distinction between NMIBC and MIBC. Grade- and subtype-specific associations were not examined because these variables were not prespecified imaging endpoints and the cohort was not powered for pathological subgroup analyses. The staging analyses were secondary and should not be interpreted as a formal head-to-head validation of complete CEUS and MRI scoring systems.

## 5. Conclusions

Real-time CEUS showed significantly higher sensitivity than the MRI inchworm sign for detecting histologically confirmed fibrovascular stalks (95.8% versus 75.0%). CEUS specificity was numerically higher than MRI specificity (90.5% versus 81.0%), but the paired difference was not statistically significant (difference, +9.5 percentage points; exact compatible 95% CI, −15.1 to +33.6; *p* = 0.625). This comparison should be interpreted in light of the different operational definitions of the two signs: CEUS positivity was based on dynamic pedicle-first enhancement, whereas the MRI inchworm sign additionally required preservation of the underlying muscular layer. This definitional asymmetry may have contributed to the lower observed MRI sensitivity and precludes interpreting the finding as evidence of a general superiority of CEUS over MRI. The stalk was strongly associated with NMIBC but did not exclude detrusor invasion. CEUS therefore adds dynamic information about papillary tumor perfusion, whereas mpMRI-based VI-RADS remains central to comprehensive assessment of muscle invasion. The high interobserver agreement supports the reproducibility of these imaging assessments among experienced readers, although broader multicenter validation involving readers with different levels of experience remains necessary. The findings support the vascular stalk as a candidate component of structured CEUS reporting and future CEUS-based staging systems, but not the replacement of MRI by CEUS on the basis of this study alone.

## Figures and Tables

**Table 1:** Patient, lesion, and histopathological characteristics. Categorical data are presented as number of patients with percentages in parentheses; continuous variables are presented as median and interquartile range (IQR).

Characteristic	Value
Patients/lesions	69/69
Men	45 (65.2%)
Women	24 (34.8%)
Age, median (IQR), years	67 (66–73)
Smoking history	51 (73.9%)
Lesion diameter, median (IQR), cm	2.5 (1.5–4.3)
NMIBC	45 (65.2%)
MIBC	24 (34.8%)
Histological fibrovascular stalk present	48 (69.6%)

**Table 2:** Diagnostic performance for detecting a histological fibrovascular stalk.

Measure	CEUS Vascular Stalk Sign	MRI Inchworm Sign
Sensitivity	95.8% (85.7–99.5)	75.0% (60.4–86.4)
Specificity	90.5% (69.6–98.8)	81.0% (58.1–94.6)
Positive predictive value	95.8% (85.7–99.5)	90.0% (76.3–97.2)
Negative predictive value	90.5% (69.6–98.8)	58.6% (38.9–76.5)
Accuracy	94.2% (85.8–98.4)	76.8% (65.1–86.1)

**Table 3:** Paired CEUS and MRI findings according to histological stalk status.

Paired Imaging Result	Stalk Present, *n*/N (%)	Stalk Absent, *n*/N (%)	Total, *n*/N (%)
CEUS positive/MRI positive	35/48 (72.9%)	1/21 (4.8%)	36/69 (52.2%)
CEUS positive/MRI negative	11/48 (22.9%)	1/21 (4.8%)	12/69 (17.4%)
CEUS negative/MRI positive	1/48 (2.1%)	3/21 (14.3%)	4/69 (5.8%)
CEUS negative/MRI negative	1/48 (2.1%)	16/21 (76.2%)	17/69 (24.6%)
Total	48	21	69

**Table 4:** Paired comparison of overall CEUS impression and VI-RADS 4–5 for detecting muscle-invasive bladder cancer. CEUS, contrast-enhanced ultrasound; CI, confidence interval; MIBC, muscle-invasive bladder cancer; VI-RADS, Vesical Imaging Reporting and Data System. Paired differences were calculated as CEUS minus VI-RADS. Confidence intervals for paired differences were calculated using the exact melded method of Fay and Lumbard and are compatible with the central two-sided exact McNemar tests. Discordant counts represent lesions correctly classified only by CEUS and lesions correctly classified only by VI-RADS, respectively. *p* values were calculated using two-sided exact McNemar tests.

Measure	CEUS, % (95% CI)	VI-RADS 4–5, % (95% CI)	Paired Difference CEUS−VI-RADS, Percentage Points (95% CI)	CEUS Correct Only/ VI-RADS Correct Only	McNemar *p*
Sensitivity	66.7 (44.7–84.4)	87.5 (67.6–97.3)	−20.8 (−42.2 to +0.9)	0/5	0.063
Specificity	88.9 (75.9–96.3)	68.9 (53.4–81.8)	+20 (+5.1 to +34.6)	9/0	0.004
Accuracy	81.2 (69.9–89.6)	75.4 (63.5–84.9)	+5.8 (−6.4 to +18.0)	9/5	0.424

**Table 5:** VI-RADS category distribution by histopathological stage.

VI-RADS Category	NMIBC	MIBC	Total
2	23	1	24
3	8	2	10
4	8	6	14
5	6	15	21
Total	45	24	69

## Data Availability

The anonymized data supporting the findings of this study are available from the corresponding author upon reasonable request, subject to applicable institutional and ethical requirements. The data are not publicly available because they include clinical imaging and histopathological information that may carry a residual risk of patient re-identification.
